# Impact of RNA Degradation on Viral Diagnosis: An Understated but Essential Step for the Successful Establishment of a Diagnosis Network

**DOI:** 10.3390/vetsci5010019

**Published:** 2018-02-06

**Authors:** Damarys Relova, Liliam Rios, Ana M. Acevedo, Liani Coronado, Carmen L. Perera, Lester J. Pérez

**Affiliations:** 1 Centro Nacional de Sanidad Agropecuaria (CENSA), OIE Collaborating Centre for Diagnosis and Risk Analysis of the Caribbean Region, La Habana 32700, Cuba; drelova@censa.edu.cu (D.R.); acevedo@censa.edu.cu (A.M.A.); lcoronado@censa.edu.cu (L.C.); claura@censa.edu.cu (C.L.P.); 2 Reiman Cancer Research Laboratory, Faculty of Medicine, University of New Brunswick, Saint John, NB E2L 4L5, Canada; lrios@unb.ca; 3 Dalhousie Medicine New Brunswick, Dalhousie University, Saint John, NB E2L 4L5, Canada

**Keywords:** viral diagnosis, RNA storage, secondary structures, laboratory networks, sample exchange, real time RT-PCR

## Abstract

The current global conditions, which include intensive globalization, climate changes, and viral evolution among other factors, have led to an increased emergence of viruses and new viral diseases; RNA viruses are key drivers of this evolution. Laboratory networks that are linked to central reference laboratories are required to conduct both active and passive environmental surveillance of this complicated global viral environment. These tasks require a continuous exchange of strains or field samples between different diagnostic laboratories. The shipment of these samples on dry ice represents both a biological hazard and a general health risk. Moreover, the requirement to ship on dry ice could be hampered by high costs, particularly in underdeveloped countries or regions located far from each other. To solve these issues, the shipment of RNA isolated from viral suspensions or directly from field samples could be a useful way to share viral genetic material. However, extracted RNA stored in aqueous solutions, even at −70 °C, is highly prone to degradation. The current study evaluated different RNA storage conditions for safety and feasibility for future use in molecular diagnostics. The in vitro RNA-transcripts obtained from an inactivated highly pathogenic avian influenza (HPAI) H5N1 virus was used as a model. The role of secondary structures in the protection of the RNA was also explored. Of the conditions evaluated, the dry pellet matrix was best able to protect viral RNA under extreme storage conditions. This method is safe, cost-effective and assures the integrity of RNA samples for reliable molecular diagnosis. This study aligns with the globally significant “Global One Health” paradigm, especially with respect to the diagnosis of emerging diseases that require confirmation by reference laboratories.

## 1. Introduction

The most recent emerging and re-emerging transboundary animal diseases (TADs) have been an important concern in animal health [1]. Examples include current reports to the World Organisation for Animal Health (Office International des Epizooties, OIE, Paris, France) regarding several outbreaks of classical swine fever (CSF) in Latvia in November 2012 [2], porcine reproductive and respiratory syndrome (PRRS) in Switzerland in November 2012 [3], highly pathogenic avian influenza (HPAI) H7N3 in Mexico in June 2012 [4], and H5N1 in Asian and African Regions that has contributed to several human cases and even deaths [5].

These examples clearly implicate RNA viruses as key drivers behind the emergence of new variants of existing pathogens. Importantly, RNA viruses have been shown to evolve with the highest mutation rates. Therefore, one of the most important tasks in diagnostic laboratories is to conduct research on the genetic information of these viral agents. This information could be used for viral classification [6], to track the spreading of epidemiological outbreaks [7], and to develop novel diagnostic methods for the detection of new viral variants [8].

The establishment of viral sequence databases has undoubtedly helped to advance the diagnosis and control of these viral diseases. Likewise, several collaborative networks have been created including OFFLU (World Organisation for Animal Health (OIE) and the Food and Agriculture Organization of the United Nations (FAO) network of expertise on avian influenza), EPIZONE (International network of veterinary research institutes working on epizootic animal diseases) and CaribVET (The Caribbean Animal Health Network). With the goal of integrating the scientific knowledge acquired among the different research laboratories, these networks improve the preparedness, prevention, detection and control of epidemics and contribute to the harmonization and reinforcement of animal disease surveillance.

Nevertheless, the exchange of viral isolates or field samples remains the critical point in the collaboration among these different diagnostic laboratories. The virus-containing clinical samples must be stored and transported at temperatures as low as possible, preferably below −70 °C. Storage at this temperature is unreliable (power failures, evaporation of liquid nitrogen or dry ice). Additionally, some viruses such as classical swine fever virus (CSFV) or foot and mouth disease virus (FMDV) can be transmitted directly by animal contact, and indirectly via contaminated equipment, foodstuff or aerosols (reviewed in [9]). Hence, shipment on dry ice represents both a biosafety hazard and a general health risk due to the infectious potential of the material. In addition, the requirement to ship on dry ice could be hampered, particularly in underdeveloped countries or regions located far from each other, due to its prohibitive costs. (reviewed in [9]).

To overcome the issues associated with the current methods of shipping unstable and infectious material, a method to effectively transport RNA isolated from viral suspensions or directly extracted from the field samples could be a useful mechanism for sharing viral genetic material between different diagnostic laboratories. However, extracted RNA stored in aqueous solutions, even at −70 °C, is prone to degradation [10]. Therefore, protecting RNA using various RNA safe solutions has been proposed [9,11,12]. The current study was designed to evaluate, different conditions used to effectively and safely store RNA for future downstream applications such as molecular diagnosis. Additionally, the role secondary structures play in the protection of RNA was explored.

## 2. Materials and Methods

### 2.1. Ethics Statement

International standards for animal welfare were used for all animal samples collected, following the regulations of the Institute of Veterinary Medicine (IMV), Ministry of Agriculture (MINAGRI) of the Republic of Cuba. The protocol # PNO-G-040-2016 of the Centro Nacional de Sanidad Agropecuaria (CENSA) was approved by the Committee on the Ethics of the MINAGRI of the Republic of Cuba and all efforts were made to minimize suffering of the animals. The samples were sent directly from the IMV to the Animal Virology Laboratory at the National Center for Animal and Plant Health (CENSA). The IMV is the official regulatory body of the Republic of Cuba; therefore, additional permits were not required.

### 2.2. Virus Strain and Matrix Samples

The inactivated isolate A/chicken/Egypt/1709-6/2008 (kindly provided by the Istituto Zooprofilattico Sperimentale delle Venezie, Padova, Italy), an HPAI (A/H5N1) virus, was used for all experiments in this study. The virus was re-suspended in 1 mL of sterile phosphate buffered saline (PBS) following the directions of the supplier.

Samples of tracheal swabs from chickens free of avian influenza virus (AIV) were collected for use as dilution matrix. The birds sampled were previously tested for AIV following the procedure described by Spackman et al. [13]. The tracheal swabs samples were pooled (10 birds/swab pool) and 1 mL PBS was added in each pool. Samples were vigorously agitated by vortexing and stored at −70 °C until RNA extraction.

### 2.3. RNA Isolation

Total RNA was obtained from the viral suspension of A/chicken/Egypt/1709-6/2008 inactivated strain and the AIV-free pooled tracheal swabs samples using QIAamp Viral RNA Mini Kit (Qiagen GmbH, Hilden, Germany) according to manufacturer’s instructions. In all cases, a starting volume of 150 µL of either tracheal swab samples or the viral suspension was used, and RNA was eluted in 50 µL nuclease free water (Promega, Madison, WI, USA). The eluted RNA was stored at −80 °C until further use.

### 2.4. In Vitro Transcription Reaction

Different RNA concentrations were fixed (high RNA load: 10^6^ copies/µL, medium RNA load: 10^4^ copies/µL and low RNA load: 10^2^ copies/µL) for in vitro-transcribed RNAs of M and H5 genes. The target regions selected were: (i) the target region reported by Spackman et al. [13] for M gene detection, which has been widely used for the diagnosis of influenza type A virus and (ii) the region reported by Lee et al. [14] for subtyping H5 from AIVs. Thus, the primer pairs M+25f/M-124r targeting M gene [13] and H155f/H699r targeting H5 gene [14] were used. In both cases the T7 promoter was coupled to the reverse primers (M-124r and H699r).

Total RNA obtained from the inactivated H5N1 strain was amplified by RT-PCR using the One Step RT-PCR Kit (Qiagen, Hilden, Germany) following the manufacturer’s directions. The amplified products were checked by 2% agarose gel electrophoresis, stained with ethidium bromide (0.5 g/mL) and purified using QIAquick gel extraction kit (Qiagen, Hilden, Germany). The RT-PCR purified products were directly in vitro transcribed from the T7 polymerase promoter site using a MEGAscript^®^ Kit (Ambion, Carlsbad, CA, USA) according to the manufacturer’s recommendations. Finally, the RNA transcripts were quantified by spectrophotometry. In this way, a total of 4.2 × 10^13^ RNA copies/µL for each transcribed gene was obtained. Standard curves based on RNA copy numbers were generated and reaction efficiencies were calculated by the LightCycler software Version 4.05 (Roche Diagnostics, Manheim, Germany). The linear ranges for both genes spanned within 10^7^–10^0^ gene copies/µL in terms of RNA copy number.

### 2.5. cDNA Synthesis and Quantitative Real-Time RT-PCR (qRT-PCR)

In all cases, the synthesis of cDNA was performed by random priming and using M-MLV reverse transcriptase, as previously described in Díaz de Arce et al. [15]. To quantify the number of copies of each gene, two different SYBR Green-based qRT-PCR assays were performed. All qRT-PCR reactions were performed in triplicate and conducted on the LightCycler^®^ 2.0 (Roche Diagnostics, Manheim, Germany) instrument. The quantification of H5 gene was performed using the qRT-PCR assay described in Pérez et al. [8].

The quantification of M gene was performed using the published primers targeting the M gene of type A influenza virus, namely forward primer M+25 and reverse primer M-124 [13], which were used for qRT-PCR based on SYBR Green I detection [16]. The final conditions of the assay were 1 µL of each forward and reverse primers (final concentration of each, 0.4 µM), 2 µL of Fast Start DNA Master SYBR Green I (10×), 1.6 µL of MgCl_2_[l2 (final concentration 3 mM), 5 µL of template and enough nuclease-free water (Promega, Madison, WI, USA) for a final volume of 20 µL for each reaction. 

### 2.6. RNA Stability Conditions

Fixed RNA loads were stored in different matrices at different temperatures for long-term analysis of RNA degradation. First, the RNA loads were set by spiking in in vitro-transcribed RNA described above into three sets of storage matrices. The different storage matrices consisted of: (i) the pooled elution obtained from samples of tracheal swabs AIV-free (ii) a modified RNA safe buffer (RSB) previously described in Hoffmann et al. [11] and (iii) RNA dry pellet. The RSB was modified substituting the RNase free water with the pooled solution obtained from the elution of the tracheal swabs AIV-free samples.

In all cases, the RNA elution obtained from the tracheal swabs AIV-free pooled samples was used for the preparation of all matrices, since it contains residual protein material and other substances normally found in clinical samples. Thus, the model uses effectively recapitulated a real field sampling case. Dry pellet was prepared adding 52.6 µL of precipitation solution (20 mg/mL Glycogen and Ammonium acetate 7.4 M in RNase free water) to 20 µL of eluted RNA of tracheal swabs from chicken.

In vitro-transcribed RNAs were serially diluted 10-fold in each of the three matrices. Three different concentrations (high, medium, and low) of each in vitro-transcript were used for the experiment. Concentrations of 10^6^, 10^4^, and 10^2^ copies/µL for each gene were finally fixed. To assess the impact of the temperature on the different storage conditions, spiked matrices were stored in parallel at 4 °C, 20 °C and 37 °C, this last condition was considered to simulate a more extreme temperature situation. These three subsets of matrices were analyzed after 3, 7, 10, 14, 21, 27, 41 and 57 days. The RNA was quantified in terms of RNA copy number by qRT-PCR. For intra-assay variability, each dilution was analyzed in triplicate.

### 2.7. RNA Secondary Structure for M and HA Genes

The secondary structures of RNA for matrix (M) and hemagglutinin (HA) genes were obtained using the RNAfold program included in the Vienna RNA software package version 2.0.0 (Institute for Theoretical Chemistry, University of Vienna, Austria) [17]. The sequences A/Goose/Guangdong/1/96, A/avian/New York/Sg-00372/2001 and A/parrot/CA/6032/04 were used to obtain a consensus secondary structure for both M (accession number: AF144306, CY048770 and DQ256384) and H5 (accession number: AF144305, CY036365 and DQ256383) genes. These sequences were selected as representative sequences for strains of the three main lineages of H5 subtypes (Eurasian, American and the emergent lineage B from Mexico) described in Perez et al. [8] The alignments of sequences obtained for both M and H5 genes were used as template. The folding of secondary RNA structures was computed based on the parameters of minimum free energy (MFE), equilibrium base-pairing probabilities, and the partition function (PF). In addition, a pattern of pair-conservation was also computed. When PF folding was selected, base-pairing probabilities were visualized in form of two Postscript dot plots, 2D graph [18].

### 2.8. Analysis Degradation Sensitibity

To assess the stability of M and HA genes under direct action of RNase, the RNA of the inactivated viral strain A/chicken/Egypt/1709-6/2008 was incubated with RNase A dilution (Promega, Madison, WI, USA). Thus, 150 µL of viral RNA were mixed with 4.5 µL of RNase A dilution (4 mg/mL diluted 1:1000) and incubated at 37 °C for different time periods (0, 30 s, 1 min, 2 min, 3 min, 4 min, 5 min, 6 min and 7 min). The integrity of the diagnostic target regions was assessed by qRT-PCR.

### 2.9. Statistical Analysis

The means and standard deviations (SD) of the RNA copy numbers were generated using the LightCycler^®^ Software 4.05.415 (Roche Applied Science, Mannheim, Germany). Significant differences between the means of RNA concentration (high, medium and low) stored in each matrix or at each temperature were analyzed by a one-way analysis of variance (ANOVA) using GraphPad Prism version 6.0 software for Windows (GraphPad Software, La Jolla, CA, USA). A Bartlett’s test was used. A *p* value < 0.05 was considered statistically significant. 

## 3. Results

### 3.1. Stability of the RNA Transcripts for M Gene Stored under Different Conditions

**Figure 1:**
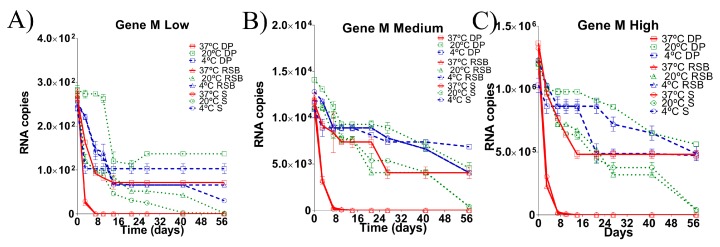
Comparison of RNA load values detected from M gene diagnostic target region under different storage conditions for 57 days post incubation (dpi). Dynamics of degradation at (**A**) low RNA load, (**B**) medium RNA load and (**C**) high RNA load. Each matrix of storage is denoted (S: swab matrix, RSB: RNA safe buffer described by Hoffmann et al. (2006) modified in this study, DP: dry pellet matrix), each temperature of storage is also denoted. Data is mean of qRT-PCR from triplicate samples. Based on the limit of detection of the assay, samples with ct > 35 were considered negative (see Supplementary Material Figure S1A).

On day 0 (t = 0 days), M gene copies ranged from 2.4–2.8 × 10^2^, 1.0–1.2 × 10^4^ and 1.2–1.3 × 10^6^ gene copies/µL, for low, medium and high RNA load, respectively, in all three storage matrices (Figure 1). The slight differences found between the estimated and the fixed concentrations (1.0 × 10^2^, 1.0 × 10^4^, 1.0 × 10^6^ gene copies/µL for low, medium and high RNA load, respectively) could reflect differences in the methods used to obtain these concentrations. For the fixed RNA loads, the RNA transcripts were quantified by spectrophotometry, whereas, at t = 0 days, the RNA load estimated by was quantified using qRT-PCR, which is based on a fluorometric method. Nevertheless, it is important to highlight that although at t = 0 the RNA load estimated was slightly higher than expected, the magnitude order for the three conditions remained in the desired range.

The target region of the M gene at low RNA load was detected by qRT-PCR at each indicated time point in all matrices stored at 4 °C (Figure 1A). However, at a temperature of 20 °C, the RNA isolated from the swab matrix was only reliably detected until 27 days post incubation (dpi); the target region of the RNA stored in RSB was detectable until 42 dpi, whereas in dry pellet matrix the RNA was stable until the end of the experiment (57 dpi) (Figure 1A). At a temperature of 37 °C, the RNA was quickly degraded in both the swab matrix or the RSB since the target region was undetectable in these storage conditions on day 8. However, for RNA stored in the dry pellet matrix, the target region of M gene was stable until the end of the experiment (57 dpi) (Figure 1A).

As with low RNA loads, the target region of the M gene was detected by qRT-PCR in all storage matrices at 4 °C until the end of the experiment when starting with a medium RNA load. The concentrations of the RNA isolated from the swab matrix and the RNA stored in RSB decreased one order (1Log) in comparison with the RNA stored in dry pellet matrix, which was stable until the end of the experiment (57 dpi) (Figure 1B). At a temperature of 20 °C, the RNA isolated from the swab matrix was reliably detected until 27 dpi, the RNA stored in RSB was detectable until 42 dpi, and the RNA stored in the dry pellet matrix was stable until the end of the experiment (57 dpi). At 37 °C, the target regions of the RNA isolated from the swab matrix and the RNA stored in RSB were undetectable by day 8. However, in dry pellet matrix, the RNA was stable until the end of the experiment (57 dpi) (Figure 1B).

Similar trends were observed when starting with high RNA loads. The M gene was detected by qRT-PCR in all storing matrices at 4 °C until the end of the experiment, although the concentrations of the RNA isolated from the swab matrix and the RNA stored in RSB decreased 1Log (Figure 1C). When stored at a temperature of 20 °C, the RNA isolated from the swab matrix was reliably detected until 27 dpi, the RNA stored in RSB was detectable until 42 dpi, and the RNA stored in the dry pellet matrix was stable until the end of the experiment (57 dpi) (Figure 1C). At a temperature of 37 °C, as with low and medium RNA loads, the target regions of the RNA isolated from the swab matrix and the RNA stored in RSB were undetectable by day 8. However, in the dry pellet matrix, the RNA was stable until the end of the experiment (57 dpi) (Figure 1C).

### 3.2. Stability of the RNA Transcript for HA Gene Stored under Different Conditions

**Figure 2:**
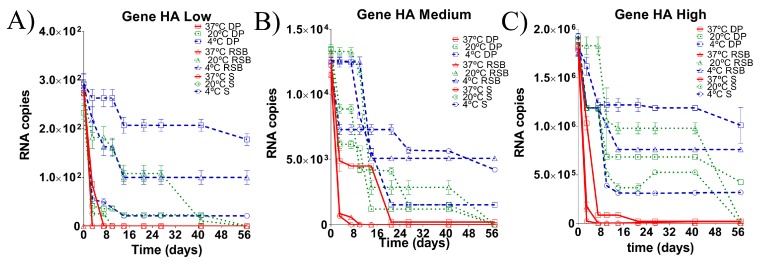
Comparison of RNA load values detected from the HA gene diagnostic target region under different storage conditions for 57 days post incubation (dpi). Dynamic of degradation at (**A**) low RNA load, (**B**) medium RNA load and (**C**) high RNA load. Each matrix of storage is denoted (S: swab matrix, RSB: RNA safe buffer described by Hoffmann et al. (2006) modified in this study, DP: dry pellet matrix), each temperature of storage is also denoted. Data is mean of qRT-PCR from triplicate samples. Based on the limit of detection of the assay, samples with ct > 35 were considered negative (see Supplementary Materials Figure S1B).

As for the M gene diagnostic target, the copy number of the HA gene (H5 subtype) was evaluated after RNA storage in different matrices and different temperatures. As for the M gene, at the beginning of the experiment (t = 0 days), the estimated RNA load was slightly higher than expected, ranging from 2.3–2.8 × 10^2^, 1.2–1.3 × 10^4^ and 1.7–1.9 × 10^6^ gene copies/µL, for low, medium and high RNA load, respectively, for all the conditions (Figure 1). However, the order of magnitude for the three estimated RNA loads remained within the desired range. The target region of the HA gene at low RNA loads was detected by qRT-PCR in all matrices at 4 °C, but the RNA load detected in the dry pellet matrix was higher than the RNA loads detected in either the RSB and swab matrix (Figure 2A). At a temperature of 20 °C, the RNA isolated from the swab matrix was only barely detected until 7 dpi, while the RNA stored in either RSB or in dry pellet was detectable until 42 dpi. Importantly, the RNA concentration in dry pellet matrix was significantly higher than in RSB at this temperature (Figure 2A). At 37 °C, the target region was undetectable either in the RNA isolated from the swab matrix or the RNA stored in RSB. For RNA stored in the dry pellet matrix, the target region of the HA gene was detectable until at least 3 dpi (Figure 2A).

For medium RNA loads, the target region of the HA gene was detected by qRT-PCR in all matrices at 4 °C until the end of the experiment (Figure 2B). At a temperature of 20 °C, the RNA isolated from the three storing matrices was reliably detected until 42 dpi (Figure 2B). At 37 °C the target regions of the RNA isolated from the swab matrix and the RNA stored in RSB were undetectable, whereas RNA stored in the dry pellet matrix was stable until the end of the experiment (57 dpi), albeit at very low concentrations (Figure 2B). 

At high RNA loads, the target region of the HA gene was detected by qRT-PCR in all three matrices at 4 °C until the end of the experiment. However, the RNA load from the swab matrix decreased one order (1Log), whereas the RNAs stored in RSB and dry pellet matrix were stable until the end of the experiment (57 dpi) (Figure 2C). At a temperature of 20 °C, the RNA isolated from the swab matrix was reliably detected until 42 dpi, whereas the RNAs stored in RSB and in dry pellet matrices were stable until 57 dpi. However, the concentration of the RNA stored in the dry pellet matrix was higher than the RNA stored in the RSB matrix (Figure 2C). At 37 °C, the target region of the RNA isolated from the swab matrix was undetectable, whereas detection of the target region from RNA stored in RSB was reliable until 42 dpi. The RNA stored in the dry pellet matrix was stable until the end of the experiment at 57 dpi (Figure 2C).

### 3.3. Secondary RNA Structures Analysis for M and H5 Genes

**Figure 3:**
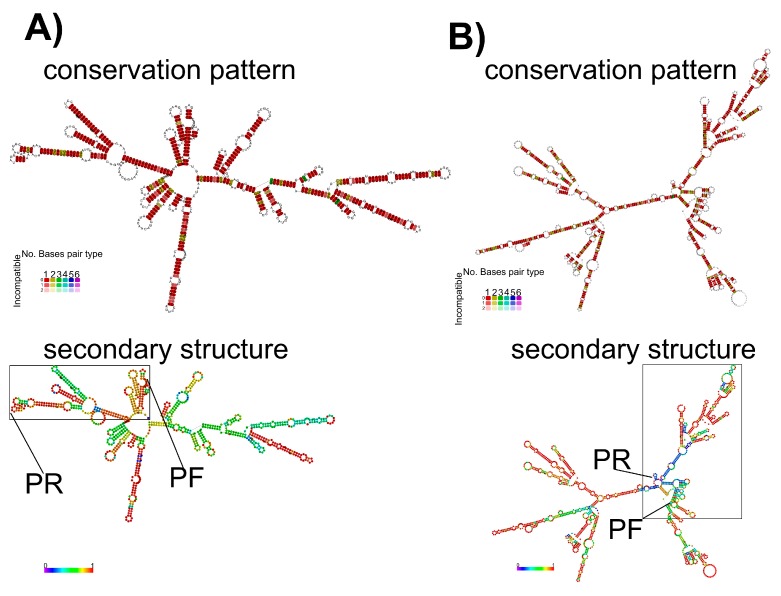
Comparison of the probability of folding for secondary structures. (**A**) Secondary structure folding and conservation pattern of pairing for M gene. The target region framed by M+25 and M-124 primers is denoted by a rectangle, and the hybridization region for each primer is highlighted. (**B**) Secondary structure folding and conservation pattern of pairing for HA gene. The target region framed by H155f and H699r is denoted by a rectangle and the hybridization region for each primer is highlighted. In the upper panels, the color scale indicates level of conservation and in the lower panels the bar indicates the scale of probability for folding.

The results obtained from the RNAfold program showed that the pattern of conservation of the secondary structure for both HA (H5 subtype) and M genes was highly conserved when strains from the most divergent H5 lineages were compared. In addition, this study also showed that the H5 gene had a higher probability to fold secondary structures when compared to the M gene (Figure 3). The RNA structure from the HA gene also showed lower global values of minimal free energy (MFE) compared to the RNA structure from the M gene (Supplementary Figures S2 and S3). However, within the diagnostic target region selected for each gene, and specifically the regions where the primers match, the RNA structure from M gene showed higher MFE and probability values to fold secondary structures in comparison to the HA gene (Figure 3). From this analysis, the regions matched by M+25 and M-124 primers were stem-loops (Figure 3A). The stem-loop shaped by the region matched by the M-124 primer had a high probability value (Figure 3). However, the regions matched by H155f and H699r primers had very low probability to shape secondary structures (Figure 3B). 

### 3.4. Evaluation of the Stability of the Diagnostic Target Regions for M and H5 Genes 

**Figure 4:**
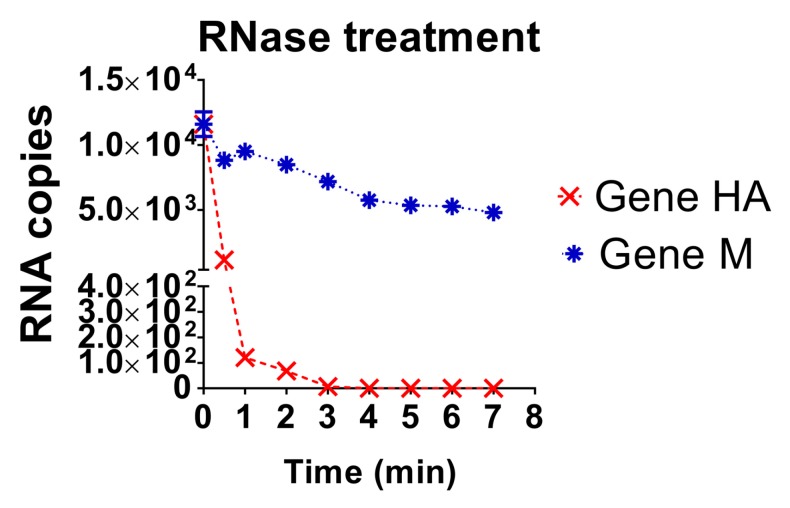
Comparison of the RNA load values under RNase A treatment for HA and M genes. The dynamics of degradation of RNA by the action of RNase A for each gene (HA gene and M gene) is shown. Data is mean of triplicate samples.

The stability of the diagnostic target regions for M and HA genes of AIV was assessed by direct action of RNase A and confirmed by quantification of RNA loads (Figure 4). The target region for the HA gene was detected until 2 min after treatment, with a sudden drop of RNA load; whereas the target region of the M gene was detected until the end of the experiment, 7 min after RNase A treatment (Figure 4). Thus, the diagnostic target region of the M gene was more resistant to RNase treatment compared to the diagnostic target region of the HA gene. 

## 4. Discussion

Current global factors, which include intensive globalization, climatic changes, and viral evolution, among others, have increased the emergence of viruses and new viral diseases over the last several decades [19]. Significantly improving diagnosis and disease control, in line with “The Global One Health” paradigm, is a challenge for veterinary services. To improve both diagnosis and disease control, there is a need for the development of laboratory networks linked to central reference laboratories that are associated with active and passive environmental and medical surveillance [20]. This concept has not yet been fully realized [21]. However, four key capacity-building requirements have been identified for its implementation. These include (i) the development of adequate science-based risk management policies, (ii) skilled-personnel capacity building, (iii) accredited veterinary and public health diagnostic laboratories with a shared database, and (iv) improved use of existing natural resources and implementation [20]. Focusing on the third initiative, aiming at accrediting veterinary diagnostic laboratories, several steps must be fulfilled by these candidate diagnostic laboratories [22]. All of these steps rely on interchangeable laboratory results based on the principles of standardization and harmonization (reviewed in [23]). Still, a critical issue affecting these diagnostic laboratories is the exchange of strains or field samples [24,25].

The current study evaluated different RNA storage conditions using RNA transcripts obtained from an inactivated HPAI H5N1 virus as model. The goal of this study was to propose a method to safely transport RNA to different laboratories for reliable downstream applications used in molecular diagnosis. Additionally, this study explore how secondary structures may affect the stability of RNA. 

The H5N1 virus was selected as model after considering several factors. First, H5N1 virus is an emergent pathogen with an RNA genome for which genetic surveillance programs are necessary in several countries. Therefore, it represents a good example where sample exchange is necessary between different diagnostic laboratories within the global surveillance network [26]. In addition, techniques capable of addressing the rapid and accurate identification and subtyping of influenza viruses are required. This means that different target genes from the same viral strain are needed with diagnostic purposes [27]. For these reasons, the H5N1 virus was selected as a model for this study.

Likewise, the RNA concentrations were selected (10^2^, 10^4^, 10^6^ copies/µL) based on the fact that they are similar to those found in a natural outbreak caused by influenza viruses [28]. From these concentrations, low, medium and high viral loads, respectively, were selected. The temperature values selected (4 °C, 20 °C and 37 °C) correspond to different environmental conditions experienced in different regions around the world such as the Caribbean and African regions, among others. Thus, the experimental conditions explored in the present work simulated real-world situations that diagnostic laboratories may face during the exchange of infectious material such as field samples and viral strains.

RNA instability is due to RNA’s high susceptibility to hydrolysis. Intracellular enzymes such as ribonucleases (RNase, endo- and exonucleases) are involved in the degradation of RNA in all living organisms [29]. Clinical samples are particularly subject to RNA degradation by the action of host nucleases [30]. The main challenge for RNA preservation is to prevent degradation by the nuclease, which co-precipitate with the nucleic acid during the purification process [31]. RNA is also vulnerable to oxidation under extreme conditions such as extended periods of retention and elevated temperatures [32]. Therefore, optimal storage conditions must be identified that can avoid RNA degradation and, in particular, also maintain RNA integrity of the diagnostic target region [33]. Successful RNA storage will ensure greater accuracy of the results obtained in subsequent molecular studies [34].

Results from this study show that the stability of the M and HA RNA transcripts depended on the temperature, matrix of storage and nature of the gene. Even though at higher RNA loads the target region was detected for most of the conditions established (except 37 °C stored in either swab matrix or RSB), the degradation curves were similar for each starting RNA concentration. Therefore, this result suggests that the RNA degradation rate, in the model studied, was independent of substrate concentration.

In terms of optimal storage temperature, the best results of stability over time were obtained at 4 °C, at which all samples tested were detectable at the end of the experiment. This outcome is not surprising given that RNase’s enzymatic activity is known to be lower at this temperature. However, this RNA storage condition can be achieved only by freezing or cooling samples for transportation and comes with all the associated drawbacks related to these methods of preservation as previously discussed (reviewed in [11]).

The only matrix able to protect the RNA from the more extreme conditions (37 °C, RNase activity optimal temperature) was the dry pellet matrix. Similar results were obtained by Martinez et al. [32], who were able to detect dried RNA after storage at 37 °C for a 2-week period [32]. It is important to highlight that, in this study, these authors also used a commercial product (GenTegra™, Pleasanton, CA, USA) for the stabilization of purified RNA before the drying process, increasing the cost of the method [32].

Nevertheless, for low concentration of the HA gene incubated at 37 °C, the RNA was detectable only 3 dpi, whereas the M gene, under the same conditions, was detected until the end of the experiment. These results suggest that the nature of each viral gene differentially affects its rate of degradation. Exploring RNA quality to perform gene expression analysis, Opitz et al. [33] found that degradation of RNA varied among different genes, especially when very short genes were compared with very long genes, where the latter was affected less [33]. However, in our study, the length of the HA transcript was larger than that of the M transcript. This suggests that additional factors, such as the stability of secondary structures, contribute to the difference in the RNA stability observed between the M and HA gene transcripts.

The prediction of secondary structures for the diagnostic target region of the M gene revealed a higher probability of folding compared to the diagnostic target region of the HA gene. In addition, the diagnostic target region of the M gene was more resistant to the RNase A treatment than the diagnostic target region of the HA gene. These results show that secondary structures are important characteristics in the nature of RNA, protecting it from degradation. Therefore, the ability of a target region to fold into secondary structures should be considered for during the diagnostic design. Currently, several thermostable reverse transcriptase enzymes have been developed to prevent failures during reverse transcription reactions caused by secondary structures. In addition, the use of chemical reagent such as 2% DMSO or 2.5 M betaine are known to improve the detection of GC-rich regions [35]. Therefore, concerns regarding the amplification of targets rich in secondary structure can be minimized.

The main limitation of our study is that the RNA model used to ensure a reliable quantification of the RNA degradation process was based on RNA in vitro transcripts instead of complete viral genes. Nevertheless, the use of clinical material (elution from negative AIV-free swabs) for the preparation of all matrices assessed, resembles the real environment during sampling process. In addition, the results obtained from this study provide evidence that the RNA degradation process was independent of its length; therefore, similar results could be expected using the complete viral genes. Despite this limitation, our study has contributed to the literature describing a practicable and cost-effective method to exchange samples between different laboratories.

## 5. Conclusions

In this study, a rigorous assessment of different conditions to store RNA safely and effectively for its downstream applications was performed. Based on our results, the optimal storage matrix is the dry pellet matrix. This storage condition was able to protect the RNA-transcript even under more extreme conditions of storage including higher temperatures and longer periods of time. Therefore, this method is proposed as the method of choice for nucleic acid material exchange between different laboratories. The importance of secondary structures within the diagnostic target regions that prevent degradation of RNA was also highlighted and should be considered in the design of novel target regions. These results point to a method that is safe, cost-effective, and ensures RNA quality for reliable molecular diagnostics. These results have global significance, particularly regarding the development of diagnostic networks for emerging diseases to maintain active and passive environmental and medical surveillance.

## Supplementary Materials

The following are available online at http://www.mdpi.com/2306-7381/5/1/19/s1, Figure S1: Analytical sensitivity and limit of quantification for SYBR Green qRT-PCR assays for specific detection of M and H5 genes of avian influenza virus. Amplification and standard curves of serial dilutions (10-fold) of in vitro-transcribed RNA for (**A**) M gene and (**B**) H5 gene of AI H5N1 subtype virus. The standard curve shows the linear range of the SYBR Green-based qRT-PCR regarding the detectable RNA. Standard curves were generated by plotting the threshold cycle (ct) values against the copy number of RNA transcripts. Figure S2: Analysis of secondary structure for M gene from a consensus alignment of influenza A virus. (**A**) Structure annotated alignment for RNA sequences of M gene from the strains A/Goose/Guangdong/1/96, A/avian/New York/Sg-00372/2001 and A/parrot/CA/6032/04 representative of the three main lineage (Eurasian, American and the new emergent lineage B). (**B**) Mountain plot representation of the MFE structure, the thermodynamic ensemble of RNA structures, and the centroid structure for the consensus structure. The positional entropy for each position are also presented. Figure S3: Analysis of secondary structure for H5 gene from a consensus alignment of influenza A virus. (**A**) Structure annotated alignment for RNA sequences of H5 gene from the strains A/Goose/Guangdong/1/96, A/avian/New York/Sg-00372/2001 and A/parrot/CA/6032/04 representative of the three main lineage (Eurasian, American and the new emergent lineage B). (**B**) Mountain plot representation of the MFE structure, the thermodynamic ensemble of RNA structures, and the centroid structure for the consensus structure. The positional entropy for each position are also presented.
