# Supplementary material for: Impact of RNA Degradation on Viral Diagnosis: An Understated but Essential Step for the Successful Establishment of a Diagnosis Network
Source: Vet Sci. 2018 Feb 6;5(1):19. doi: 10.3390/vetsci5010019 (PMC5876574; doi:10.3390/vetsci5010019)
Supplement: Supplementary file 1 [file vetsci-05-00019-s001.pdf]

Supporting information

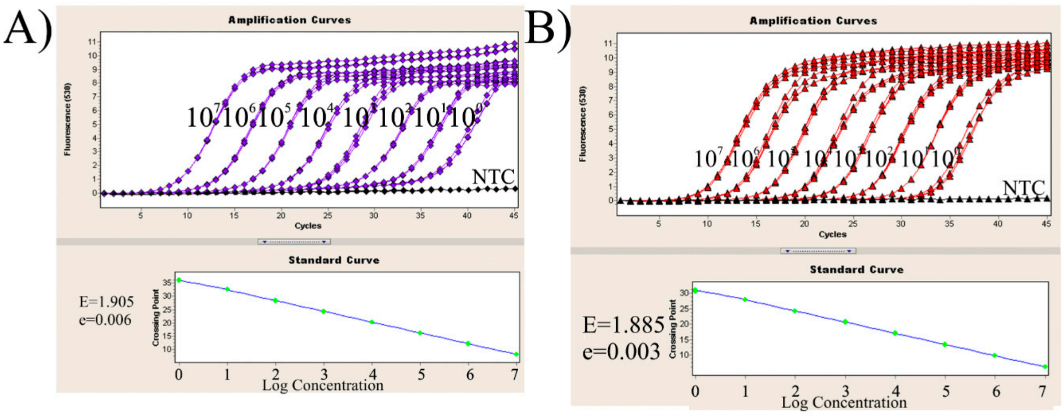

Figure S1. Analytical sensitivity and limit of quantification for SYBR Green qRT-PCR assays for specific detection of M and H5 genes of avian influenza virus. Amplification and standard curves of serial dilutions (10-fold) of in vitro-transcribed RNA for (**A**) M gene and (**B**) H5 gene of AI H5N1 subtype virus. The standard curve shows the linear range of the SYBR Green-based qRT-PCR regarding the detectable RNA. Standard curves were generated by plotting the threshold cycle (Ct) values against the copy number of RNA transcripts.

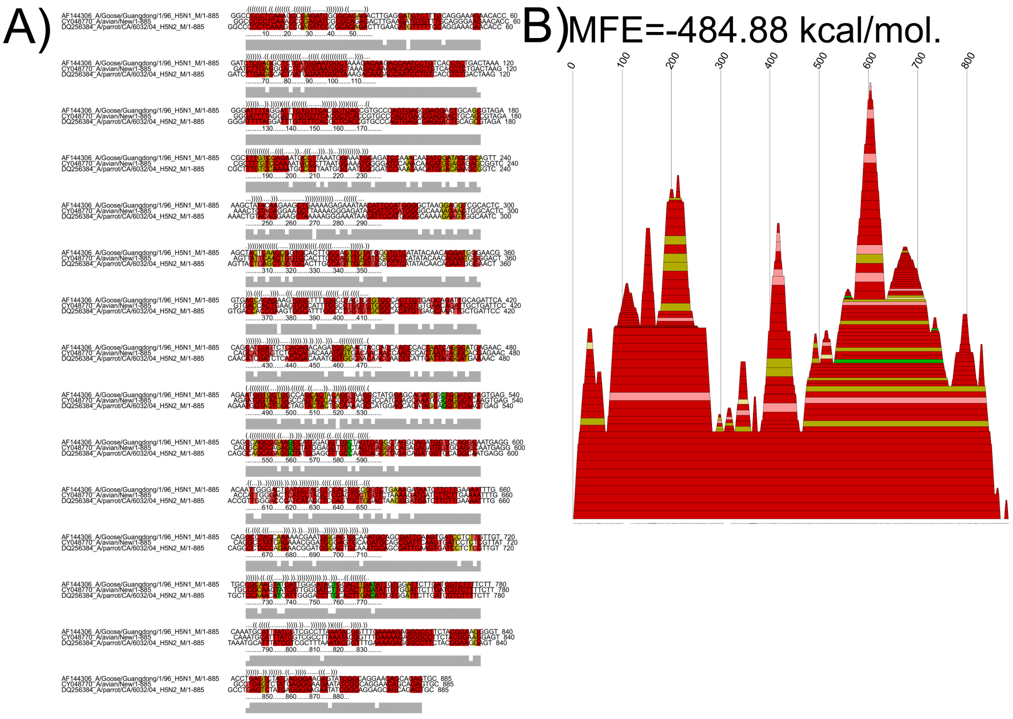

Figure S2. Analysis of secondary structure for M gene from a consensus alignment of influenza A virus. (**A**) Structure annotated alignment for RNA sequences of M gene from the strains A/Goose/Guangdong/1/96, A/avian/New York/Sg-00372/2001 and A/parrot/CA/6032/04 representative of the three main lineage (Eurasian, American and the new emergent lineage B). (**B**) Mountain plot representation of the MFE structure, the thermodynamic ensemble of RNA structures, and the centroid structure for the consensus structure. The positional entropy for each position are also presented.

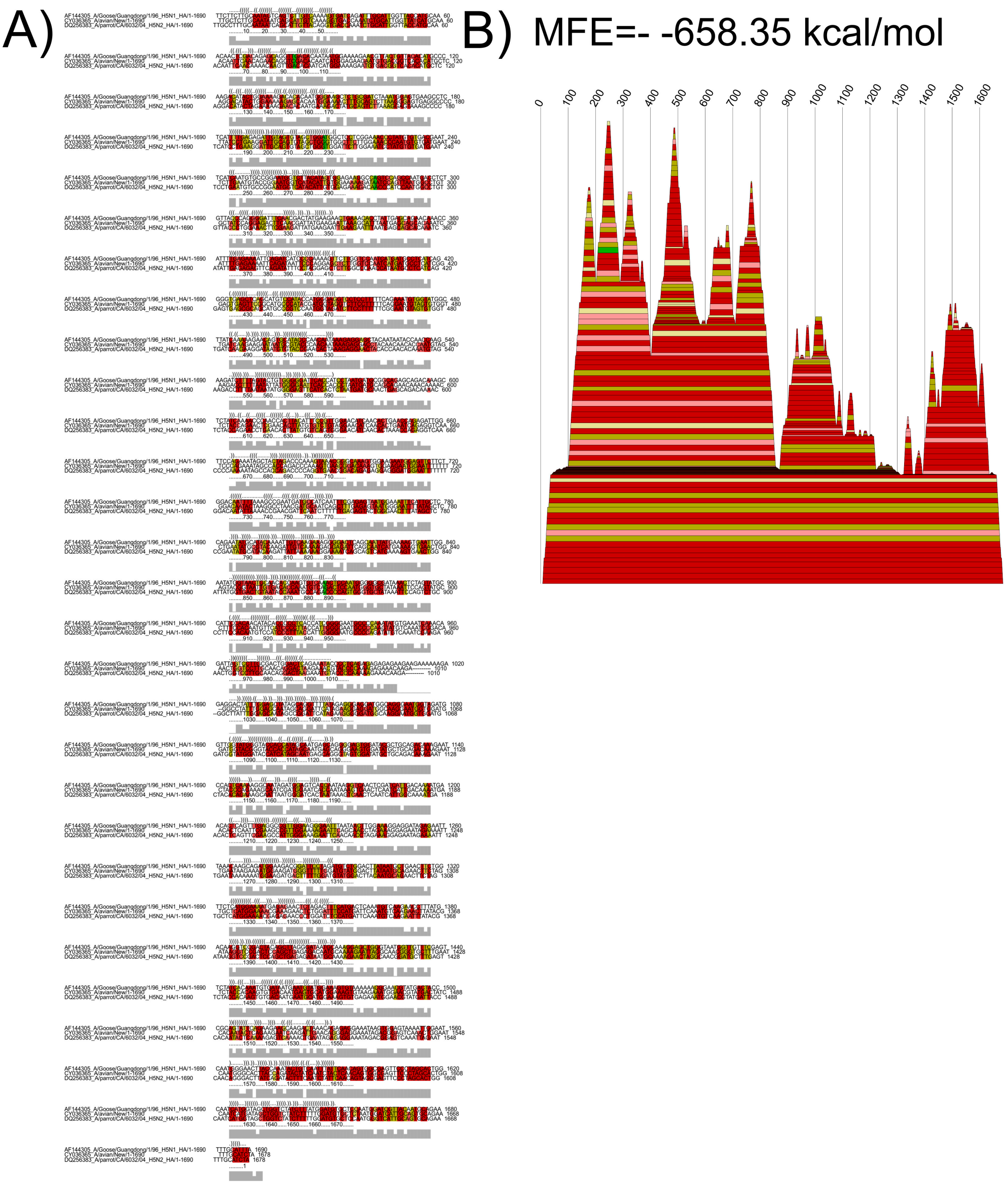

Figure S3. Analysis of secondary structure for H5 gene from a consensus alignment of influenza A virus. (**A**) Structure annotated alignment for RNA sequences of H5 gene from the strains A/Goose/Guangdong/1/96, A/avian/New York/Sg-00372/2001 and A/parrot/CA/6032/04 representative of the three main lineage (Eurasian, American and the new emergent lineage B). (**B**) Mountain plot representation of the MFE structure, the thermodynamic ensemble of RNA structures, and the centroid structure for the consensus structure. The positional entropy for each position are also presented.
